# Inflammatory Reaction as Determinant of Foreign Body Reaction Is an Early and Susceptible Event after Mesh Implantation

**DOI:** 10.1155/2014/510807

**Published:** 2014-03-26

**Authors:** Holger Gerullis, Evangelos Georgas, Mihaly Borós, Bernd Klosterhalfen, Christoph Eimer, Christian Arndt, Stephan Otto, Dimitri Barski, Dirk Ysebaert, Albert Ramon, Thomas Otto

**Affiliations:** ^1^Department of Urology, Lukas Hospital, Preussenstraße 84, 41464 Neuss, Germany; ^2^German Centre for Assessment and Evaluation of Innovative Techniques in Medicine (DZITM), Germany; ^3^Department of Experimental Surgery, University of Szeged, Hungary; ^4^German Centre for Implant-Pathology, Düren, Germany; ^5^Department of Surgery, Clemens Hospital Münster, Germany; ^6^Department of Surgery, Universitair Ziekenhuis, Antwerpen, Belgium; ^7^ITERA (International Tissue Engineering Research Association), Belgium

## Abstract

*Purpose.* To investigate and relate the ultrashort-term and long-term courses of determinants for foreign body reaction as biocompatibility predictors for meshes in an animal model. *Materials and Methods.* Three different meshes (TVT, UltraPro, and PVDF) were implanted in sheep. Native and plasma coated meshes were placed bilaterally: (a) interaperitoneally, (b) as fascia onlay, and (c) as muscle onlay (fascia sublay). At 5 min, 20 min, 60 min, and 120 min meshes were explanted and histochemically investigated for inflammatory infiltrate, macrophage infiltration, vessel formation, myofibroblast invasion, and connective tissue accumulation. The results were related to long-term values over 24 months. *Results.* Macrophage invasion reached highest extents with up to 60% in short-term and decreased within 24 months to about 30%. Inflammatory infiltrate increased within the first 2 hours, the reached levels and the different extents and ranking among the investigated meshes remained stable during long-term follow up. For myofibroblasts, connective tissue, and CD31+ cells, no activity was detected during the first 120 min. *Conclusion.* The local inflammatory reaction is an early and susceptible event after mesh implantation. It cannot be influenced by prior plasma coating and does not depend on the localisation of implantation.

## 1. Introduction

A mesh when implanted for a particular indication represents a foreign body which induces a foreign body reaction (FBR). This reaction is triggered by the initial acute phase reaction and the subsequent construction of the implant matrix, mostly conducted by migration of fibroblasts producing glycosaminoglycans and collagen. The FBR has been histologically described as a foreign body granuloma adjacent to the mesh fiber and a surrounding collagen capsule that shields the host from the foreign material. It seems likely that such a chronic inflammatory process impairs normal wound healing and tissue regeneration and may result in reduced functionality and increased side effects when clinically applied [1]. However, the process of FBR does not necessarily reduce the proposed mesh function of restoring mechanical functionality in a particular region of the body. Several attempts have been conducted to improve biocompatibility of meshes and reduce FBR [2–4]. The exact FBR mechanisms and respective time flow* in vivo* are not entirely understood. In a previously conducted large animal long-term experiment over 24 months, applying different meshes and investigating their biocompatibility course, it could be shown that the FBR had been already established after 3 months and did not significantly change over 24 months [5]. Those results lead to the assumption that FBR is being determined early in the* in vivo* course. In addition, in this previous study the investigated meshes maintained constant positions when ranking their biocompatibility characteristics and comparing it to their respective* in vitro* performances. Aim of this current study was therefore to investigate the early extent of macrophage invasion and infiltration of inflammatory and connective tissue as determinants of FBR immediately after mesh implantation and to compare those values to the respective intermediate and long-term performances.

## 2. Material and Methods

### 2.1. Animal Studies

We conducted this animal experiment in accordance with the National Institutes of Health guidelines (Guide for the Care and Use of Laboratory Animals). The experimental protocol was approved by the Animal Welfare Committee at the University of Szeged, Hungary (license/permission number V01353/2010). The entire animal experiment has been divided into a long-term study investigating biocompatibility over 24 months as previously published and this current study. The long-term experiment has been previously described and included 14 animals [5].

**Figure 1:**
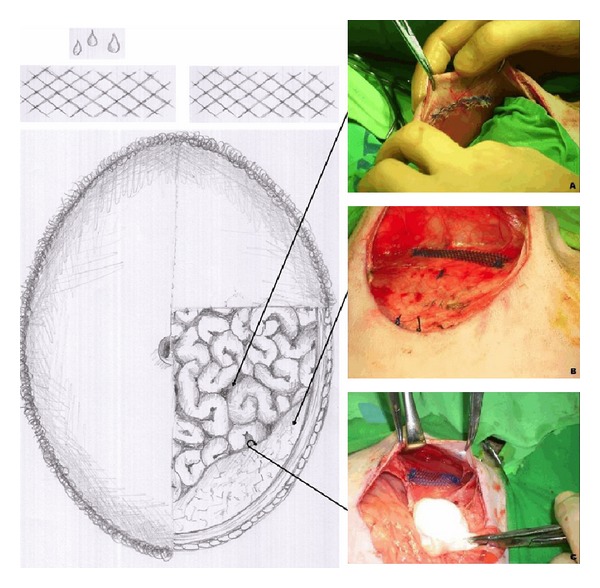
Intra-operative situs during implantation: (A) intraperitoneal, (B) fascia onlay, (C) muscle onlay. 12 hours prior to implantation meshes were coated and incubated with autologous plasma. Meshes were implanted bilaterally into the torso to allow intraindividual comparison of coated versus uncoated meshes per animal.

For the short-term study, additional three female sheep, weighing from 20 to 25 kg and at least 6 months old, were included. Housing and veterinary care were provided at Szeged University's farm for experimental animal studies. We previously selected three meshes characterised as good (PVDF), intermediate (UltraPro), and poor (TVT, Tension-free vaginal tape, polypropylene)* in vitro* and* in vivo* performer according to a recently developed test system and* in vivo* long-term evaluation [5, 6]. Surgery was performed following the protocol as previously described [5]. After a longitudinal laparotomy, we implanted meshes in 3 different locations, bilaterally: (a) interaperitoneally, (b) as fascia onlay, and (c) as muscle onlay (fascia sublay) (Figure 1). The size of the implanted meshes was 3 × 12 cm. Meshes were fixed with two sutures at both ends. Mean operation time for the implantation was 50 min. For every native mesh implant, a respective plasma-coated version was implanted in equivalent localizations on the contralateral site of the torso. For this purpose, meshes had been incubated with autologous plasma at least 12 hrs prior to implantation. In the current study, we selected one sheep per investigated mesh resulting in 3 animals. Chosen time points for explantation were 5 min, 20 min, 60 min, and 120 min. At every explantation time point, we dissected a piece of about 3 × 4 cm size from the initially implanted mesh.

### 2.2. Morphological Studies

A single mesh section (3 × 4 cm) and adhesive tissue were obtained from each explanted mesh. Tissue samples were fixed in 10% formalin, then sliced into 0.3 × 1 cm pieces, and embedded in paraffin. Each 10 to 15 sections of 4 *μ*m thickness were stained with haematoxylin and eosin (H&E), as well as periodic acid Schiff (PAS) plus diastase and Elastica van Gieson (EvG). All mesh specimens were studied by light microscopy (LM). LM was controlled by immunohistochemistry which was performed on the material embedded in paraffin using the avidin-biotin complex method with diaminobenzidine as a chromogen. The procedure was repeated twice for every sample. Antibodies used in this study included polyclonal rabbit anti-human CD3, 1 : 50 as pan marker for T-lymphocytes (DAKO, Hamburg, Germany), polyclonal rabbit anti-human CD138, 1 : 50 as pan marker for plasma cells (DAKO, Hamburg, Germany), monoclonal mouse antiporcine CD68, 1 : 50 (DAKO, Hamburg, Germany) as pan marker for macrophages, monoclonal anti-human CD15, 1 : 10 (Becton Dickinson, Heidelberg, Germany) as marker for polymorphonuclear granulocytes (PMNs), polyclonal rabbit antiactin protein, 1 : 200 (DAKO, Hamburg, Germany), and monoclonal anti-CD34 1 : 200 (BIOMOL, Hamburg, Germany) as markers for fibromyocytes as well as monoclonal porcine CD31, 1 : 10 (DIANOVA, Hamburg, Germany) as marker for endothelial cells. The morphometric evaluation consisted of a quantitative cell analysis of the inflammatory reaction and soft-tissue reaction. The cells were counted each in 5 HE-slides in 10 fields at a grid of 10 points (100x, area 0.1 mm^2^) and in the interface (0–300 mm, 400x, area 625 mm^2^). Parameters measured were the inflammatory infiltrate (*μ*m), connective tissue (*μ*m), vessels (PV%), macrophages (%), leukocytes (%), polymorphonuclear granulocytes (PMNs, %), and fibroblasts (%) as well as TUNEL, Ki67, and HSP 70 expressing cells (%).

### 2.3. Statistics

The influence of the clinical data on the tissue response was tested for significance by performing an ANOVA with LSD-modification according to Bonferroni as an established method for comparative experiments in which only the difference in outcomes is of interest. Statistical significance was assumed at *P* < 0.05.

## 3. Results

**Figure 2:**
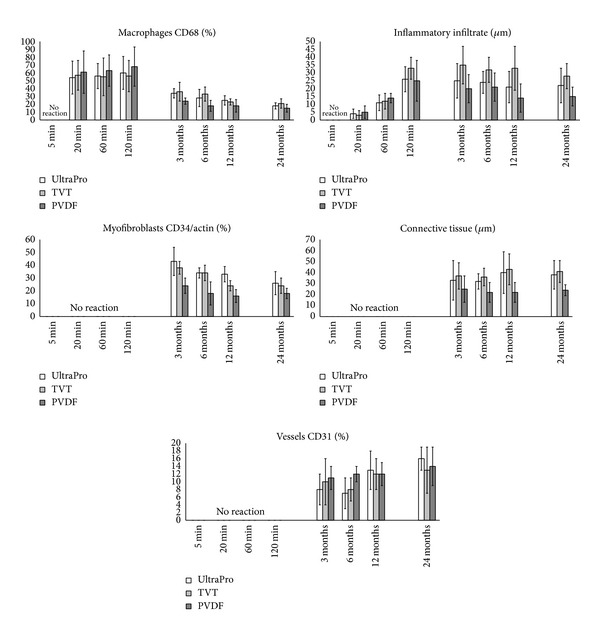
Time course of investigated biocompatibility markers relating ultra short term results to long term results (over 24 months).

Overall we did not see minor nor major complications during surgery. In addition, no macroscopic differences among the native and plasma coated meshes immediately after explantation were remarked. As in previous studies, main focus was set on parameters measured for inflammatory infiltrate, connective tissue, macrophages (CD68), endothelial cells as markers for vascularisation, and myofibroblasts when microscopically investigating the different mesh reactions after explantation. High extent of connective tissue reaction and inflammatory reaction was assumed as indicative for reduced biocompatibility.  Figure 2 graphically demonstrates and relates the short-term and long-term course of 5 important markers for early FBR determination.

Within the first two hours after implantation, an early invasion of macrophages at comparable extent in all meshes culminating after 120 min was observed. The induced inflammatory reaction expressed by the extent of inflammatory infiltrate revealed the same trend but increased slowly. Macrophage invasion was detectable after 20 min at a relatively high level of about 50% and slightly increased up to 70%. Interestingly, the macrophage invasion was highest in the PVDF meshes, which in the long-term approach performed best with lowest chronic inflammation. The respective early inflammatory infiltrate continuously increased within the first 60 min in all investigated meshes. However, after 120 min, this trend inverted in the PVDF meshes. In contrast, in the TVT and UltraPro, the inflammatory infiltrate was still increasing towards 120 min. Not surprisingly, no connective tissue was observed after 120 min. Additionally measured endothelial cells representative for vessel integration and myofibroblasts were all negative during the initial 120 min after implantation.

Two markers, representative for early FBR signs, did show relevant activity within the first two hours after mesh implantation. Thus for those markers, inflammatory tissue, and macrophage invasion, a comparison of the coated versus uncoated version of the respective meshes was possible but did not show relevant differences.

Each mesh (coated and uncoated) was placed and investigated in three different positions of the torso (Figure 1). Neither in the short-term nor in the long-term approach, we observed differences regarding the reaction of the FBR determinants on the meshes. In addition, plasma coating did not have an influence regarding the mesh performance in the different regions of the body.

## 4. Discussion

In this animal study we investigated* in vivo* biocompatibility predictors for three different meshes by measuring early and long-term signs of foreign body reaction (FBR) as macrophage invasion, inflammatory reaction, and connective tissue determination at the implant site of the meshes. Comparing these results to the long-term data in the same species (sheep), we can show that the process of determination of FBR is defined early in the course after implantation for markers of local acute inflammation. In contrast, myofibroblast invasion, vascularisation, and connective tissue adhesion are not relevantly presented in the ultrashort-term course. The extent of macrophage invasion and inflammatory tissue does not relevantly increase after 120 min compared to the values for 3 months after explantation or later. A previously described method to improve biocompatibility performance of meshes* in vivo* and* in vitro* by autologous plasma coating before implantation did not have an effect on early inflammatory events as the respective values for inflammatory infiltrate and macrophage invasion did not differ from coated to native meshes [5–7]. However, markers like connective tissue organisation, myofibroblast invasion, and endothelial cells, characteristic for vascularisation, are detectable after 3 months after implantation and show different extents in the three investigated meshes.

To the best of our knowledge, this current and the previous study display the longest combined short- and long-term* in vivo* approach for investigating biocompatibility issues on meshes* in vivo*. In addition, so far, no ultrashort-term investigations* in vivo* have been reported as most of the currently available studies investigated effects on meshes earliest after 7–21 days [8]. It has been shown that, at 7 days after implantation of a mesh, an acute inflammatory reaction occurs, dominated by macrophage invasion [9]. Over time, this early inflammatory process transforms into a chronic, at times granulomatous reaction, promoting wound healing but also forming small granulomas [10]. It is known that the extent of collagen formation may vary during this process, whereas a severe inflammatory reaction, with disordered fibrin and collagen deposition, is likely to compromise the integration process and functional outcome. When investigating prolene and a porcine dermal collagen implant (Pelvicol), Zheng and colleagues could show a first acute phase reaction after 48 hours peaking at day 7–14. Our data adds ultrashort-term information suggesting that this reaction is starting even earlier in the course, after minutes. Zheng and coworkers described the acute reaction to diminish and finally reach negligible levels by 90 days which can be partially supported in our current study.

Foreign body reactions to alloplastic mesh material are primarily induced by inflammatory cells like macrophages and T-lymphocytes [11, 12]. Macrophages have a critical role in acute inflammation and early vascularisation, as well as in the subsequent chronic phase of the host response as they are known to be capable of differentiating towards two pathways. This M1/M2 polarisation enhances macrophages leading to an immediate and/or persistent inflammation or leading to a constructive remodelling and new tissue generation [13]. However, this polarisation has not been investigated in the current study. When considering wound healing, it is known that CD68-positive macrophages reach their maximum level on second day after injury and slowly decline afterward [14]. We show that high percentages of CD68 positive macrophages are detectable on the meshes after minutes and hours already. Our current data does not contradict this statement of Engelhardt and coworkers, and in contrast, it motivates to more specifically confine the process of the first acute inflammatory reaction. This could be of interest when investigating and developing mesh modification strategies to influence this early acute reaction. A previously developed plasma coating strategy to optimize biocompatibility of meshes seems not to influence this early inflammatory reaction and inflammatory infiltrate formation but more so to influence mid- and long-term processes which lead to neovascularization, collagen fibre organisation. It has been shown that premature type III collagen is predominantly synthesized in early phases of wound healing and in the presence of inflammatory cells [15]. Collagen III is then replaced by highly cross-linked and stable collagen type I later after implantation. Delayed wound healing, immature scar development due to persistent chronic inflammation, may be predicted by a lowered collagen type I/III ratio [16, 17]. A favourable type I/III collagen ratio is known to improve biocompatibility and can be positively influenced by preimplantation mesh modifications, for instance, gentamicin coating [18]. When considering the results of the present study, an increase in inflammatory infiltrate was shown in all three meshes. After 120 min PVDF could be shown to increase considerably slower than TVT and UltraPro. Although hypothetically, the previously shown good long-term biocompatibility performance of PVDF may be triggered by an early decrease of the acute inflammatory reaction and subsequent modification of the meshes microenvironment leading to, for example, improved collagen I/III ratio, we did not observe a direct influence of the localisation of the implanted mesh in the body in the short- nor long-term study being reproducible in the coated and uncoated meshes. Although the localisations have been chosen to cover different structural parts of the body with different immunologic potential and physical/mechanic strain, localisation seems not to be of outmost importance for the biocompatibility performance of a mesh* in vivo*.

This study has limitations. Only three meshes have been investigated; thus a higher number of analysed meshes would have supported the findings. Meshes have been chosen based on previously obtained data from* in vitro* and* in vivo* approaches. However, those* in vitro* studies investigating biocompatibility features of randomly selected meshes have been validated* in vivo* supporting the presented approach. To our knowledge, this experiment represents the first a combined ultrashort- and long-term study for biocompatibility features of native and modified meshes and provides interesting data for the current understanding of FBR determination and FBR course* in vivo*.

## 5. Conclusion

The local inflammatory reaction is an early and susceptible event after mesh implantation. It cannot be influenced by prior plasma coating and does not depend on the localisation of implantation.

## Abbreviations

FBR: Foreign body reaction
PVDF: Polyvinylidene fluoride
CD: Cluster of differentiation
TVT: Tension-free vaginal tape.
